# A Foreground Prototype-Based One-Shot Segmentation of Brain Tumors

**DOI:** 10.3390/diagnostics13071282

**Published:** 2023-03-28

**Authors:** Ananthakrishnan Balasundaram, Muthu Subash Kavitha, Yogarajah Pratheepan, Dhamale Akshat, Maddirala Venkata Kaushik

**Affiliations:** 1 School of Computer Science and Engineering, Vellore Institute of Technology, Chennai 600127, Tamil Nadu, India; balasundaram.a@vit.ac.in (A.B.);; 2 School of Information and Data Sciences, Nagasaki University, Nagasaki 852-8521, Japan; 3 School of Computing, Engineering and Intelligent System, Ulster University, Londonderry BT48 7JL, UK; p.yogarajah@ulster.ac.uk

**Keywords:** magnetic resonance imaging, few-shot learning, foreground prototypes

## Abstract

The potential for enhancing brain tumor segmentation with few-shot learning is enormous. While several deep learning networks (DNNs) show promising segmentation results, they all take a substantial amount of training data in order to yield appropriate results. Moreover, a prominent problem for most of these models is to perform well in unseen classes. To overcome these challenges, we propose a one-shot learning model to segment brain tumors on brain magnetic resonance images (MRI) based on a single prototype similarity score. With the use of recently developed few-shot learning techniques, where training and testing are carried out utilizing support and query sets of images, we attempt to acquire a definitive tumor region by focusing on slices containing foreground classes. It is unlike other recent DNNs that employed the entire set of images. The training of this model is carried out in an iterative manner where in each iteration, random slices containing foreground classes of randomly sampled data are selected as the query set, along with a different random slice from the same sample as the support set. In order to differentiate query images from class prototypes, we used a metric learning-based approach based on non-parametric thresholds. We employed the multimodal Brain Tumor Image Segmentation (BraTS) 2021 dataset with 60 training images and 350 testing images. The effectiveness of the model is evaluated using the mean dice score and mean IoU score. The experimental results provided a dice score of 83.42 which was greater than other works in the literature. Additionally, the proposed one-shot segmentation model outperforms the conventional methods in terms of computational time, memory usage, and the number of data.

## 1. Introduction

Brain tumors are one of the most dangerous tumors. The most frequent primary brain tumor is gliomas, and it comprises about 2% of all malignancies [1,2]. Over the course of a year, registrations in the neuro-oncology clinic at a tertiary care facility were thoroughly analyzed from hospital-based databases tracking CNS (central nervous system) cancers. For people aged 15 to 39, the 5-year survival rate nears 72%. In India, the prevalence of brain tumors is from 5 to 10 per 100,000 people and is on the rise. Hence, brain tumors are needed to be identified as early as possible for early treatment and recovery. In several applications of computer-aided diagnosis, medical image segmentation is crucial. The region of interest is extracted automatically without involving any manual segmentation procedures and is regarded as the most crucial step in medical imaging procedures.

The conventional structural MRI image segmentation and analysis of brain tumors is an onerous and time-consuming task.

In general, three theoretical categories are used for automated brain segmentation techniques utilizing MR images: intensity-based, machine learning, and hybrid. The highly heterogeneous appearance of brain tumors, especially sub-regions, makes segmentation difficult [3]. Furthermore, aberrations such as motion and/or field in homogeneity can make the problem even worse. There are several deep learning techniques that have already been implemented in automatic segmentation. However, these processes call for an extensive amount of training data to detect tumors thus make the process computationally expensive. CNN-based models [4,5], being the most common in deep learning architectures, use three-dimensional filters or custom filters to optimize themselves for tumor foreground regions.

State-of-the-art architectures in this field tend to be U-Net and PANet, which show the likelihood of high accuracy scores but on the downside, they use more training data. The use of multi-task networks [6] utilizes the model cascade strategy to develop a solution for class imbalances. Despite its outstanding performance, this method leads to undesired complexity in the model. The use of shared parameters to learn joint features is effective but requires a good size of training data and task-specific parameters which makes it computationally heavy to train. Ref. [6] has shown that they perform much better than other architectures (including a few-shot based) but use ensemble models and post-processing approaches, which adds time required to evaluate each image. The use of transformer-based learning is also one of the most widely used methods to extract and fuse multi-scale features. Ref. [7] has adopted a similar squeeze-and-expansion transformer at the core as a segmentation framework. Having an enormous number of parameters and loading three-dimensional images makes it very computationally inefficient to train and test. Such networks required extensive training in the model. The winner of the BraTS 2018 challenge has performed segmentation using autoencoder regularizations [8]. Although that network proved considerable results, due to its large network width, it required a heavy GPU to assess the large number of parameters.

In order to reduce the complexity of the model, we propose using a few-shot learning technique to segment brain tumors. Generally, unique and distinct classes are chosen for validation and the base data are set in the few-shot model so that the generalization performance is assessed on new classes. We are partially inspired by the state-of-the-art model by Hansen [9] to segment brain tumors. Few-shot learning is a part of meta-learning, which is making the model learn to identify the similarities between the image and support set and label the query image. As it uses only a few labeled samples to segment the query image, the training data required to perform the computation decreases significantly; thus, it reduces the number of network parameters and computational costs.

In this work, we used prototype-based few-shot learning, i.e., the support images are selected and their features are extracted and embedded in a vector space. The features of the query image are then extracted and the similarity score is calculated using the cosine score. In contrast to the conventional frameworks, our segmentation method utilizes a one-shot learning method. As far as we know, this is the first study to implement one-shot learning that uses a single prototype for modeling the foreground class for the segmentation of tumor regions from the MRI images of the brain. 

The contribution of this study can be summarized as follows:

1. Propose a one-shot learning segmentation model by considering the foreground prototypes for brain tumor detection on brain MRI that transcends the current deep learning-based tumor segmentation models in regard to the size of the training data.
2. Adopt the VGG-16-based encoder with pre-trained weights from Resnet101 trained on MS COCO datasets as our few-shot learning model.
3. Experiment with different N-shot K-way methods to show the effectiveness of the proposed single prototype for modeling the foreground classes based on its uniformity.
4. Compare the performance of the proposed approach to architectures that are used for brain tumor segmentation.
5. Evaluate the performance of our methods by using the dice similarity coefficient and intersection over union.

## 2. Related Work

### 2.1. Deep Learning-Based Brain Tumor Segmentation

Deep learning-based brain tumor segmentation methods utilize various CNN-like neural networks to segment the tumor regions, as they can occur anywhere in any shape or size and so as to automate the process. Ref. [10] has implemented a CNN-based network for glioblastoma. The method in [11] proposed to work only a small part of the image containing the tumor using a C-CNN that utilized two different paths for extracting local and global features. Ref. [12] used nested structures to tackle segmenting different image modalities. Ref. [13] has made use of BCM-CNN and utilized a sine–cosine grey fitness algorithm for tuning. The method in [14] applied U-Net to segment tumor images. It utilized an ensemble of various models with different hyper-parameters to obtain better results and reduce random errors. The method in [15] developed a 3D-Dense-UNet model to simplify complex multi-class segmentation problems. These methods do not work very well on unseen tumor classes and also take a lot of processing time as well. The method in [16] proposed a unique CNN and probabilistic neural network architecture to segment the tumor effectively. Cascaded CNNs were implemented by [17] on three tumor regions, thus implementing multiclass segmentation. However, almost all of these DNN learning models are classification-based models. A comparative analysis of existing tumor segmentation methods [18] shows that they require a large amount of training data to accurately segment the tumor images effectively, and its supervised learning performance on unseen classes is not effective. However, our one-shot-based learning can understand the similarities and differences that work well in unseen classes.

### 2.2. Self-Supervised Learning 

Self-supervised learning learns to identify one part of the input through another by eliminating the need for labels. The few-shot learning (FSL) model is a fully unsupervised model which performs semantic segmentation, and it improved the mIoU score of the previous model. The method in [19] carried out self-supervised depth estimation by utilizing diverse in-depth features and the difficulty in learning the depths. Ref. [20] predicted the position by analyzing the anatomy of cardiac MR images. Context restoration has also been used for medical image segmentation [21,22]. Our proposed model also fits the criteria of the self-supervised learning paradigm. Our model extracts the features of the query prototype and embeds them into the feature vector space where the similarity scores of the feature vector and support vector are compared. Subsequently, the query image is segmented, making the process semi-self-supervised. The support images in our model are introduced in batches and thus the model learns to find the similarity scores between the query and support images in a semi-supervised manner.

### 2.3. Few-Shot Learning

In medical-based image segmentation, fewer datasets are available. Therefore, we are using few-shot learning, a method in which the model learns to segment the query images using a small set of images which reduces the training data. It performs well on unseen classes as it works on similarity scores between support and query images. The method in [19] has stated that few-shot classification learns classifiers from just a few samples of each class that have been tagged. Multi-feature comparison utilizing two branches is also an effective mechanism for the few-shot learning model [23]. The method in [24] stated that there are two types of few-shot learning methods: inductive, in which only the support images are introduced to the classifier, and transudative, in which both support and query sets are utilized by the classifier. Model agnostic meta-learning is a technique for discovering any standard model’s parameters through meta-learning in order to prepare that model for quick adaptation [25]. Matching networks introduced by [26] have an embedded space in which the weighted nearest neighbors classifiers are applied. Prototypical neural networks revolve around developing an embedding space that points the clusters around a single prototype [27]. The method in [28] has applied few-shot learning on natural language processing very efficiently.

### 2.4. Few-Shot Segmentation

Semantic segmentation forms clusters of images which belong to the same object class at the pixel level. The method in [4] used a model which comprises two commanding modules. The first module determines if the support and query image visually correspond, while the second dictates the network to focus on the targeted query objects. The method in [29] proposed a novel strategy that automated the process of matching query prototype and query image which are obtained by high-profile query image predictions. The tumor intensity, shape, and location may differ from image to image. However, identifying them requires pixel-level classification. Utilizing both foreground and background features at the pixel level provided optimum results in implementation [30,31]. Ref. [32] proposed a one-shot image segmentation technique similar to ours. The method in [29] utilized a Gaussian mixture model to evaluate prototypical clusters of different classes. The method in [33] has implemented a few-shot-based U-Net architectures for detecting radiographic patterns and obtained an improvement in accuracy. Path aggregation network (PANet) is a unique metric learning-based model that utilizes a prototype alignment network. In the embedding space, PANet learns by extracting specific prototypes of each class from a small number of support images and segments the query images by matching each pixel to the prototypes it has learned. PANet provides high-quality prototypes that are discriminative for many classes and representative of each semantic class via non-parametric metric learning [34]. ADnet main [9] used a model and utilized the homogeneity of the foreground prototype by computing anomaly scores. It is reported to use one foreground prototype and, therefore, outperforms state-of-the-art models for abdominal organ and heart segmentation. Our model utilizes prototype-based learning using one-shot learning that is different from the aforementioned models.

## 3. Proposed Methodology

In this work, we propose a similarity-measured prototypical one-shot segmentation model to classify tumor regions in MRI images. Using a common feature extractor between query and support sets of images, metric learning-based segmentation is performed in the target domain. We solely focus on slices containing foreground classes, unlike many other recent deep learning algorithms that employ the entire set of images.

### 3.1. Problem Foundation

We aim to obtain a segmentation model trained using few-shot learning methods which can learn faster than traditional learning methods and require only a few labeled images from the same classes. In this model, images containing distinct training classes $C_{train}$ (e.g.,: $C_{train}$ = NCR/NET—label 1, ED—label 2, ET—label 4) and testing $C_{test}$ are obtained from the training and testing dataset $D_{train}$ and $D_{test}$, respectively. The segmentation model *Μ* is trained on $C_{train}$ and tested on $C_{test}$.

Taking $C_{train}$ as the training and $C_{test}$ as the testing set, the model is trained in an episodic way. Each episode consists of a group {S,Q} (a set of support images and a set of query images). These pairs of ${\left.\left\{S_{i},Q_{i}\right.\right\}}_{i=1}^{N}$, where N denotes the total number of images in the dataset, sum up the whole dataset. Considering the C-shot K-way segmentation learning task for each episode pair $\left.\left\{S_{i},Q_{i}\right.\right\}$, we obtain the output as the predicted query mask. The support set $S=\left\{\left(x_{1},y_{1}\right),..,\left(x_{C\times K},y_{C\times K}\right)\right\}$ consists of C×K number of image mask pairs {x, y}, where $x,y\in R^{X\times Y}$ are slices of each image having X×Y image dimensions. The query set Q = {$\overset{´}{x}$, $\overset{´}{y}$} contains an image mask pair with a single slice.

### 3.2. Few-Shot Learning Model

**Figure 1:**
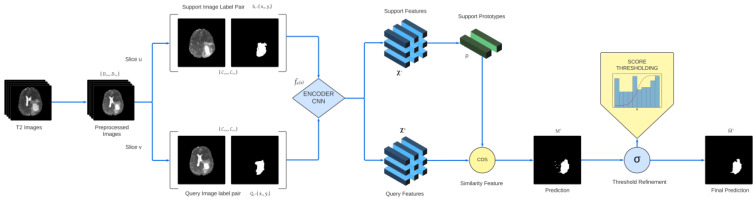
A pipeline of the proposed prototypical one-shot segmentation model to separate brain tumor regions.

Given a set of labeled images, our few-shot learning-based model aims to learn adaptation to new classes when trained under very few labeled samples. The training of the model is carried out in an iterative manner where in each iteration, random slices containing foreground classes of randomly sampled data are selected as the query set along with a different random slice from the same sample as the support set. Unlike previous methods for obtaining prototypes for classes in both the foreground and background [35,36], we are only considering the foreground prototypes. This eliminates the possibility of overtraining the large black background class. The process summarizing the model training is shown in Figure 1.

#### 3.2.1. Preprocessing and the Extraction of Prototype Features 

A brain tumor is highly heterogeneous in respect of pixel intensity, size, and location. It results in highly imbalanced data, as the number of health voxels constitutes 98% of all voxels [10]. To overcome this challenge, our model uses prototype representations of labeled classes. Before that, each T2 multi-parametric MRI (mpMRI image) and its corresponding label is first pre-processed. The bright ends of each image are cut off to alleviate the off-resonance issue. Images and labels are then resampled to unify spacing. The ROIs of each image are then cropped out according to the label to unify image sizes.

**Figure 2:**
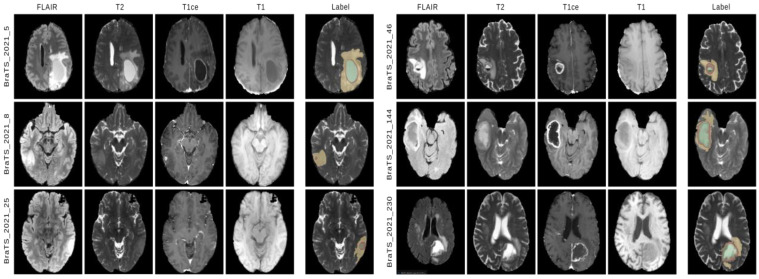
Illustration of six samples of multi-parametric MRI images (slice containing tumor region) from the BraTS 2021 dataset depicting Flair, T2, T1ce, T1 modalities, and label. The yellow, green, and red regions on the label indicate the tumor regions of different levels.

Once the dimensionality of images is reduced, the feature encoder $f_{\theta }$ extract features for both query $f_{\theta }\left(x\right):Q\to \chi^{q}$ and support images $f_{\theta }\left(x\right):S\to \chi^{s}$. As our aim is to model only the foreground class in each episode, we considered only the prototypical features of foreground class *s* to be extracted. The feature map $\chi^{q}$ is then resized to mask dimensions $\left(X,Y\right)$. Each foreground prototype ρ in embedding space is calculated using masked average pooling (MAP) for each foreground class:

(1) $$\rho =\underset{c}{\sum }\frac{\sum_{\left(i,j\right)}\chi^{q}\left(i,j\right).\left[\eta \left(i,j\right)=c\right]}{\sum_{\left(i,j\right)}\left[\eta \left(i,j\right)=c\right]}$$

where $\left(i,j\right)$ indexes the spatial locations of query slice and $\eta \left(i,j\right)$ denotes the Dirac measure which represents the almost sure probability outcome in the sample space. The Dirac measure is computed by:

(2) $$\eta_{\left(i,j\right)}\left(Z\right)=l_{Z}\left(i,j\right)=\left\{\begin{array}{c} 0,\forall \left(i,j\right)\notin Z\\ 1,otherwise \end{array}\right.\;$$

where $Z_{\left(i,j\right)}\leftarrow \left[Q^{\left(i,j\right)}=c\right]$ is a sampling function that extracts given spatial coordinates $\left(i,j\right)$ from query image *Q* having *c* foreground class. Figure 2 shows samples of multi-parametric MRI images from the dataset depicting Flair, T2, T1ce, T1 modalities, and label. The yellow, green, and red regions on the label denote the tumor regions of different levels.

#### 3.2.2. Similarity Feature-Based Segmentation 

In order to differentiate query images *c* from class prototypes, we used a metric learning-based approach based on non-parametric thresholds. This is performed using a similarity measure *R* (Tucker’s congruence coefficient) to compute similarities between foreground prototype feature class ρ and query feature $\chi^{q}$, which is computed by:

(3) $$R_{\left(i,j\right)}=-\alpha \frac{\chi_{\left(i,j\right)}^{q}.\rho }{\sqrt{{\left(\chi_{\left(i,j\right)}^{q}\right)}^{2}.\rho^{2}}}$$

where α is a scaling factor whose value is set to 20. Studies [9,34] indicated that multiplying Tucker’s congruence coefficient by α attains considerable performance compared to using Euclidean distance for similarity measures. It enables incongruent query feature vectors to receive a relative score of α. To obtain the final dense predicted mask, we performed soft thresholding with sigmoid function σ(.) along with an evaluated parameter β:

(4) $$M_{\left(i,j\right)}^{q}=1-\sigma \left(R_{\left(i,j\right)}-\beta \right)$$

This enables the query feature vectors with a similarity score below β to achieve a minimum foreground probability which is set to 0.5, whereas feature vectors above 0.5 obtain a foreground probability below β.

Having an end-to-end network to be trained in an iterative manner, where each episode $Q_{i}$,$S_{i}\;$ is taken as input. We calculated the binary cross-entropy segmentation loss as:

(5) $$L_{seg}=\frac{-1}{N}\underset{\left(i,j\right)}{\sum }\underset{p_{c}\epsilon \rho }{\sum }\left[\eta \left(i,j\right)=c\right].log{\left(M\right)}_{\left(i,j\right)}^{q}$$

where $p_{c}$ represents prototype features of class *c*. Following [34], we have also added a prototype alignment regularization loss which reverses the query and supports set predictions enabling the information to flow both ways. This greatly helps the model to align queries and support prototypes. It is computed by:

(6) $$L_{PAR}=\frac{-1}{XY}\underset{\left(i,j\right)}{\sum }\underset{p_{c}^{q}\epsilon \rho }{\sum }\left[\eta \left(i,j\right)=c\right].log{\left(M\right)}_{\left(i,j\right)}^{q}$$

where $p_{c}^{q}$ represents query features of class *c*. PAR helps the model to learn in the embedding space consistently and to align the prototypes generated from the support set.

Thus, the total loss for our model can be formulated as:

(7) $$L_{Total}=L_{seg}+\lambda L_{PAR}$$

where λ works as a controlling parameter for the effect of PAR loss. In our experiments, different values of lambda did not lead to significant improvements in score, and hence λ was kept at 1.

**Figure 3:**
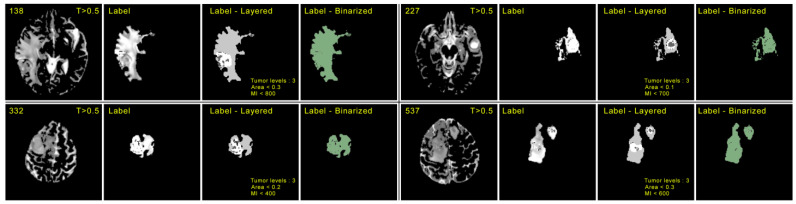
Demonstration of the procedure for determining the portion of the tumor in each slice. Here, the first column of each image contains the serial number according to the BraTS dataset and the corresponding random slice with an intensity threshold > 0.5. The second column contains the corresponding label of each scan. The third column depicts the layered structure of labels highlighting tumor levels, area, and mean intensities (MI). The fourth column contains the binarized form of label data.

The Algorithm 1 pertaining to foreground prototypes based on few-shot segmentation is shown below in Figure 3.
**Algorithm 1.** Foreground prototypes based on few-shot segmentation
**Require**: Pre-processing of images, such as identifying and cropping out ROI regions of the image.**Require**: Base feature encoder model $f_{\theta }$. 1.   Sample $D_{train}$ and $D_{test}$ from pre-processed images 2.   Initialize {$C_{train}$, $C_{test}$} empty3.   **for** Image set {$I_{train}$, $I_{test}$} in {$D_{train}$, $D_{test}$} **do**4.       cur ← 05.**       while** i = 0 to C x K **do**6.          index ← i + curr7.          Sample slice u, v from {$I_{train}$ [index], $I_{test}\left[index\right]$} 8.          $S_{u}\;\leftarrow \;\left\{x_{u},\;y_{u}\right\}$//where $x_{u}$ is T2 image and $y_{u}$ is its corresponding label
9.          $Q_{v}\;\leftarrow \;\left\{x_{v},\;y_{v}\right\}$
10.         {$C_{train}$, $C_{test}$} ← {{$S_{train},\;Q_{train}\},\left\{S_{test},\;Q_{test}\right\}\}$ ∈ {$S_{u},\;Q_{v}$} 
11.      **end while**

12.   **end for**
13.   **for**
$\left\{C_{i\_train},\;C_{i\_test}\right\}$ in {$C_{train}$, $C_{test}$} **do**
14.      $Q\;\leftarrow \left\{C_{i\_train},\;C_{i\_test}\right\}\;\in$ Query set 15.      $S\;\leftarrow \left\{C_{i\_train},\;C_{i\_test}\right\}\;\in$ Support set16.      
$\chi^{q}\;\leftarrow \;f_{\theta }\left(Q\right)$
17.      
$\chi^{s}\;\leftarrow \;f_{\theta }\left(S\right)$
18.   
**end for**
19.    **for** k = 0 to len($\chi^{s}$) **do**
20.       ℙ[k] $\leftarrow \;\rho \left(\chi_{k}^{s}\right)$ // ρ = masked average pooling function (MAP)21.    **end for**22.    **for** n = 0 to len($\chi^{q})$ **do**23.      Predictions[n] ← R(ℙ[n], $\chi_{n}^{s}$)24.      Result[n] ← σ (Predictions[n]) // σ = Threshold function to eliminate small artifacts25.   
**end for**


Here, C x K refers to C-shot K-way based segmentation. C stands for the number of classes and K for the number of samples from each class to train on. $S_{u}$ and $Q_{v}$ represents support and query sets for slice u and slice v, respectively. $\chi^{q}$ and $\chi^{s}$ are the features extracted from the query and support set, respectively. R (x,y) represents the cosine similarity between x and y objects. 

The pseudocode logic for training our few-shot segmentation model is shown in Algorithm 2.
**Algorithm 2.****Pseudocode** for training one-shot segmentation model.Define the size and dimension of images in the dataset.**Initialization**: Support set $S=\left(x,\;y\right)$, Query set Q = {$\overset{´}{x}$, $\overset{´}{y}$} contains image mask pair having a single slice.Choose a backbone architecture as a feature encoder model $f_{\theta }$ with multi-class identification having θ parameters.**For** epoch in S steps / I iterations.   **For** each image label pair in the train dataset.        Divide image label pairs into query and support sets containing random slices from the image. Both the query and support set contain one slice and its corresponding label image.       Pass both sets through the feature encoder model to extract their features.       Calculate support prototypes using masked average pooling of the support features set.        Perform segmentation based on similarities found between query features and support prototypes.       Update total loss consisting of segmentation and PAR loss. 
   **End for**
**End for**

## 4. Experimental Settings

### 4.1. Dataset

We employed the multimodal Brain Tumor Image Segmentation 2021 (BraTS) dataset [37,38,39] as a benchmark to evaluate our model. It contains 1470 NIfTI files of 3D mpMRI scans with an average of 150 slices each. Out of these, 1250 were labeled and 220 were unlabeled validation sets. Only the T2-weighted NIfTI files of each scan were used for evaluation. In pre-processing, the following steps were carried out: (1) eliminate the 0.5% intensities as an effort to reduce the sharpness in the high end of the intensity curve, (2) re-sample the number of slices to around 20, preserving the same spatial resolution, and (3) resize the image to 256 × 256 in order to standardize the dimensions. Furthermore, each slice is replicated thrice in order to accommodate the RGB channels in our network. To increase the variability of the data, image transformations (shearing, geometrical changes, rotations, translations) were applied randomly on slices before feeding them to the network without augmenting the data.

### 4.2. Parameter Settings

**Figure 4:**
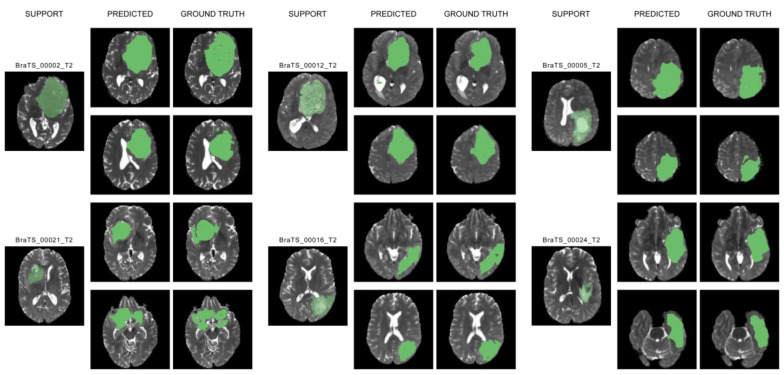
Qualitative results of our model with different support sets on two slices of different query images along with their ground truths. In these figures, tumor regions are depicted in green. The proposed learning method achieves desirable segmentation results which can be verified with their corresponding ground truth images.

We initialized the VGG-16-based encoder with pre-trained weights from Resnet101 trained on MS COCO [40]. The model is trained in an end-to-end manner with a stochastic gradient descent with a momentum of 0.9, a batch size of 1, a learning rate of 10^−3^, and a decay rate of 0.97 per 1000 epochs. Additionally, another weight decay of 5 × 10^−4^ is used over 50 k iterations. Weights are applied on foreground classes (1.0) and background regions (0.2) with cross-entropy loss to address the class imbalances. Out of 1250 MRI scans, only 60 MRI scans were used for training purposes. The training process as a whole takes 2 h on a Nvidia RTX 3060 GPU. During testing, results are averaged based on a 5-fold cross-validation of over 1000 iterations per epoch. Foreground classes are produced as a form of mask over the background region in a binarized format. as shown in Figure 3 and Figure 4.

### 4.3. Evaluation Metrics

We have utilized dice score and IOU score as metrics to evaluate our model. Dice similarity coefficient or dice score D is a reproducibility validation metric between segmentations X and Y, and is defined as:

(8) $$D\left(X,Y\right)=\frac{2*\left|X\cap Y\right|}{\left|X\right|+\left|Y\right|}$$

We used intersection over union (IoU) score to understand the ratio of the predicted segmentation area to the underlying ground truth. This helps us to understand the amount of overlap and mean average precision of our model. Precision is another valuable metric to identify the correctness of our predicted segmentation mask for foreground classes. It is calculated by dividing the number of correctly predicted tumor region pixels by the sum of correctly and incorrectly classified tumor region pixels.

### 4.4. Evaluation Protocols

In the inference stage, query scans are segmented episode-wise based on annotated support data. The position of the foreground class varies largely. Therefore, an evaluation protocol that does not depend on the target volume is required. Here, we sampled each slice from the support foreground volume and used this data to segment the whole query scan. To effectively carry out this technique, we selected a random slice from the middle of the support volume which contains a high amount of information regarding the foreground class. For effective classification between foreground and background classes, we have clustered the different levels of tumors in a single class and it is fed forward into the neural network as a singular target feature. Scores are calculated based on the accuracy of the mask generated by our model for the target region.

## 5. Results and Discussion

The experimental results obtained from our FSS model based on training on the BraTS 2021 dataset (60 training images and 350 testing images) for different N-shot K-way methods are shown in Table 1. It is observed that in practicality, increasing N and K values results in a slight-to-no increase in score metrics. This could be due to the random extraction of slices for the support set which may or may not contain many similarities with the query images. Moreover, an increasing number of sample images (N-shot) increases the potential of sampling drastically different sizes, shapes, and intensity tumor regions of different scans which can affect the score in a negative way.

The similarity between ground truth and predicted segmentation is assessed by two major comparison metrics. We used the mean and maximum dice score and mean and maximum intersection over union (IOU) score to assess our findings. The maximum metric of these scores is considered in order to quantify the extent to which our proposed one-shot segmentation proves to be effective. The performance metrics with 1-shot 1-way show a high average dice core of 83% and an average IOU of 80.9% compared to the other increasing number of samples carried out in the conventional studies. Model predictions along with their ground truths are depicted in Figure 4. 

In a typical CNN, the number of features in each feature map is the constant times the number of input pixels n (typically the constant is <1). Convolving a fixed size filter across an image with n pixels takes O(n) time since each output is just the sum product between k pixels in the image and k weights in the filter, and k does not vary with n. Similarly, any max. or avg. pooling operation does not take more than linear time in the input size. Therefore, the overall runtime is still linear.

The total trainable parameters in our few-shot model are estimated to be 59,215,000, with a size of 172 MB. This is subject to change based on the feature encoder model used. The encoding shape represents the shape of $\chi^{q}$ and $\chi^{s}$ after query set (Q) and support set (S) is passed through $f_{\theta }$, respectively. Table 1 shows the encoding shape and memory usage statistics of the proposed model. 

The experimental results obtained from several deep learning segmentation methods for brain tumor segmentation are listed in Table 2. We compared our model with these frameworks, as there are no other few-shot models for brain tumor segmentation. The OM-Net [6] resolves the imbalance problem of the tumor segmentation class but utilized more training data. Segtran [7] generated effective receptive fields but had a more complex architecture. The use of autoencoders in NVDLMED [8] for segmentation makes it more computationally expensive. In comparison to these models [41], our proposed approach contains fewer parameters and is not much computationally expensive. Furthermore, our one-shot segmentation model took a very low inference time for each image, which makes it very suitable to test unseen images in clinical settings.

Additionally, the proposed approach allows extracting the data required to classify the definition of borders between foreground and background classes, which can be trained using very few training images. This unlocks the possibilities of obtaining effective results in medical imaging applications where a sizable amount of data may not be present. Few-shot learning models have been proven to be effective in various other domains including mask aggregation [30], semantic scene segmentation [42], image classification [43], and text generation [44]. Using minimal data, the few-shot learning technique is able to achieve significant results in several scenarios. Table 3 shows the dice score results of 1 shot, 5-shot, and 10-shot for 1-way and 5-way segmentation.

The critical aspect of this study is to identify the largely homogeneous foreground class in terms of size, shape, and intensity values. This sporadically leads to arbitrary or inaccurate identification of foreground region in volumes where tumor pixel intensity (MI < 200) and the background brain region have almost similar values. Our approach of a single prototype for modeling the foreground classes is based on its uniformity. However, if this uniformity is not met, and the foreground consists of multiple distinct regions with strong edges, and then one prototype may not suffice. One option to capture multiple foreground regions during inference is to take inspiration from [45] and cluster the features into consolidate multiple foreground classes into one region.

## 6. Conclusions

In this work, we proposed a prototypical one-shot learning framework to segment brain tumors on MRI scans. The proposed method is able to extract robust prototypes and performed segmentation of the foreground class using non-parametric distance calculation. It is observed that despite using a small fraction of data, we are able to generate almost equal results compared to the other deep learning approaches. As it is a metric learning-based approach, it is not bound to be used on a particular type of MRI-scanned image. Furthermore, it is not required to set a large number of support images as, it is observed that a single support image (1-shot, 1-way) proves to be effective for segmentation. Moreover, increasing the support set size reduces the performance slightly. This may be due to differences in similarity scores obtained across various heterogeneous-sized foreground classes. Therefore, we conclude that the foreground prototype-based few-shot learning approach proposed reduces the amount of training and testing time significantly compared to conventional deep learning methods. With this model, we prove that significant results can be obtained in clinical settings where a high amount of data may not be present. Future work will be in the direction of developing a generic framework that is not bound to any specific dataset across the healthcare domain.

## Figures and Tables

**Table 1:** Encoding shape (spatial) and memory usage statistics along with a feature encoding shape of (1,5,10)—shot (1,5)—way learning models.

Method	Input Size (MB)	Forward/Backward Pass Size (MB)	Estimated Total Size (MB)	Encoding Shape
**1-shot 1-way**	17.56	21,038.63	21,228.29	(22,256,32,32)
**1-shot 5-way**	21.76	24,863.83	25,057.69	(26,256,32,32)
**5-shot 1-way**	21.76	24,863.83	25,057.69	(26,256,32,32)
**5-shot 5-way**	42.73	43,989.76	44,204.69	(46,256,32,32)
**10-shot 1-way**	27.00	29,645.34	29,844.44	(31,256,32,32)
**10-shot 5-way**	68.95	67,897.39	68,138.44	(71,256,32,32)

**Table 2:** Comparative table showing dice scores (in percentage) of different deep learning methods with the amount of training data (number of MRI scans) used along with dataset names.

Method	Training Data	Dice (%)	Dataset
OM-Net + CGap	559	91.59	BraTS 2015 + BraTS 2018
3DCNN + CRF	338	90.10	BraTS 2015 + ISLES 2015
Segtran	335	81.70	BraTS 2019
NVDLMED	285	87.04	BraTS 2018
AFN-6	274	89.30	BraTS 2015
CNN + 3D filters	274	80.00	BraTS 2015
**Few-shot-based**	**60**	**83.42**	**BraTS 2021**

**Table 3:** Results of (1, 5, 10)-shot (1, 5)-way segmentation on the BraTS 2021 dataset using mean and maximum dice and IoU metrics (in percentage). Scores are obtained from the validation of 250 randomly sampled volume datasets.

Method	Score Metrics				
	Avg. Dice	Max. Dice	Avg. IOU	Max. IOU	Precision
**1-shot 1-way**	**83.42 ± 0.35**	**83.85 ± 1.14**	80.97 ± 1.12	**81.57 ± 1.23**	**61.03 ± 0.38**
1-shot 5-way	83.78 ± 1.14	82.67 ± 2.81	**80.99 ± 1.16**	81.39 ± 1.61	60.78 ±0.82
5-shot 1-way	83.28 ± 2.27	82.50 ± 3.13	79.44 ± 2.29	80.97 ± 0.25	60.45 ± 1.05
5-shot 5-way	83.41 ± 2.30	81.23 ± 3.43	79.64 ± 2.44	80.22 ± 1.68	60.61 ± 1.75
10-shot 1-way	82.89 ± 2.78	81.45 ± 2.25	79.59 ± 2.54	80.98 ± 3.02	79.62 ± 1.68
10-shot 5-way	83.40 ± 3.28	80.23 ± 4.17	80.02 ± 2.69	80.82 ± 2.93	59.88 ± 2.19

## Data Availability

The data used to support the findings are available upon request. Additionally, the dataset can be downloaded at http://braintumorsegmentation.org/ (accessed on 2 January 2023). The code pertaining to this work is available at https://gitfront.io/r/Akshat/iCDty4sG5dpu/foreground-prototypes-based-few-shot-learning/ (accessed on 2 January 2023).
